# Pattern Recognition in the Processing of Electromyographic Signals for Selected Expressions of Polish Sign Language

**DOI:** 10.3390/s24206710

**Published:** 2024-10-18

**Authors:** Anna Filipowska, Wojciech Filipowski, Julia Mieszczanin, Katarzyna Bryzik, Maciej Henkel, Emilia Skwarek, Paweł Raif, Szymon Sieciński, Rafał Doniec, Barbara Mika, Julia Bodak, Piotr Ferst, Marcin Pieniążek, Kamil Pilarski, Marcin Grzegorzek

**Affiliations:** 1 Department of Medical Informatics and Artificial Intelligence, Faculty of Biomedical Engineering, Silesian University of Technology, Roosevelta 40, 41-800 Zabrze, Poland; anna.filipowska@polsl.pl (A.F.); julimie273@student.polsl.pl (J.M.); katabry222@student.polsl.pl (K.B.); emilskw843@student.polsl.pl (E.S.); pawel.raif@polsl.pl (P.R.); rafal.doniec@polsl.pl (R.D.); barbara.mika@polsl.pl (B.M.); julibod178@student.polsl.pl (J.B.); piotfer581@student.polsl.pl (P.F.); marcpie568@student.polsl.pl (M.P.); kp272628@student.polsl.pl (K.P.); 2 Department of Telecommunications and Teleinformatics, Faculty of Automatic Control, Electronics and Computer Science, Silesian University of Technology, Akademicka 16, 44-100 Gliwice, Poland; wojciech.filipowski@polsl.pl; 3 Faculty of Applied Mathematics, Silesian University of Technology, Kaszubska 23, 44-100 Gliwice, Poland; mh308176@student.polsl.pl; 4 Institute for Medical Informatics, University of Luebeck, Ratzeburger Allee 160, 23562 Lübeck, Germany; 5 Department of Clinical Engineering, Academy of Silesia, Rolna 43, 40-555 Katowice, Poland; 6 Łukasiewicz Research Network—Krakow Institute of Technology, The Centre for Biomedical Engeenering, Zakopiańska 73, 30-418 Kraków, Poland; 7 German Research Center for Artificial Intelligence, Ratzeburger Allee 160, 23562 Lübeck, Germany

**Keywords:** Polish Sign Language, electromyography, gesture recognition, MyoWare, BIOPAC

## Abstract

Gesture recognition has become a significant part of human–machine interaction, particularly when verbal interaction is not feasible. The rapid development of biomedical sensing and machine learning algorithms, including electromyography (EMG) and convolutional neural networks (CNNs), has enabled the interpretation of sign languages, including the Polish Sign Language, based on EMG signals. The objective was to classify the game control gestures and Polish Sign Language gestures recorded specifically for this study using two different data acquisition systems: BIOPAC MP36 and MyoWare 2.0. We compared the classification performance of various machine learning algorithms, with a particular emphasis on CNNs on the dataset of EMG signals representing 24 gestures, recorded using both types of EMG sensors. The results (98.324% versus ≤7.8571% and 95.5307% versus ≤10.2697% of accuracy for CNNs and other classifiers in data recorded with BIOPAC MP36 and MyoWare, respectively) indicate that CNNs demonstrate superior accuracy. These results suggest the feasibility of using lower-cost sensors for effective gesture classification and the viability of integrating affordable EMG-based technologies into broader gesture recognition frameworks, providing a cost-effective solution for real-world applications. The dataset created during the study offers a basis for future studies on EMG-based recognition of Polish Sign Language.

## 1. Introduction

Gesture recognition has become increasingly significant in the realm of automated systems, particularly for applications involving communication in environments where verbal interaction is hindered. This is notably critical in contexts such as emergency response, communication with individuals with speech disabilities, or the overcoming of language barriers in life-threatening situations. The rapid development of machine learning algorithms has enabled advancements in processing electromyographic (EMG) signals, offering promising avenues for the interpretation of sign languages, including Polish Sign Language (PSL).

Gesture recognition is exceptionally crucial in automatic threat detection systems, particularly in crowded areas such as airports and mass gatherings. However, in our case, there was a need to address the challenge of supporting people who do not communicate naturally. Reasons for such a circumstance may include life-threatening situations, language barriers, speech organ dysfunction, or finding yourself in a situation where communication is not feasible. The holistic expressions of the same thought assume that gestures are immediately understood, which speech gives in a hierarchical analytical form [1]. In the work of [2], credit is given to [3,4,5] for discovering the link between speech sounds and gesture movements. McNeill’s study elaborates on this insight at higher semantics and pragmatics linguistic levels. In an engineering context, we collect data that can serve as a basic communication dictionary without using speech organs, for example, through gestures. After collecting the data, there is a need and desire to obtain information and results from the collected data. This process can take many forms or algorithms, and in the case of sign language recognition, several algorithms are used to achieve this goal. EMG signals are commonly used in sign language recognition, using various approaches, while “CNN, often paired with LSTM, ranks third in classifier popularity and can achieve exceptional accuracy, reaching up to 99.6% when using both EMG and IMU data” [5]. This means that using CNN as well as LSTM yields excellent results in sign language recognition.

In another study, detailed in the article titled “A Novel Methodology for Classifying EMG Movements Based on SVM and Genetic Algorithms” [6], researchers used genetic algorithms (GA) as an optimization method and compared their performance with support vector machines (SVM), which were fine-tuned using particle swarm optimization (PSO). This comprehensive approach yielded convincing results, ultimately highlighting the superiority of genetic algorithms as a more effective method of analyzing electromyographic data [7].

The study “Evaluation of Surface EMG Features for the Recognition of American Sign Language Gestures” [8] used a different approach. They used the Mahalanobis distance criterion to assess a set of 16 features extracted from surface EMG signals. The discriminant analysis was then applied, resulting in an accuracy of 97.7%. This result underscores the robustness of the selected methodology in accurately recognizing gestures in American Sign Language based on surface EMG features [9].

Another relevant paper discusses using graph-based deep neural networks for skeleton-based sign language recognition. Although this study primarily examines the Turkish and Chinese Sign Languages, its approach could be adapted for the German Sign Language. The method focuses on extracting features from the whole-body skeleton to improve accuracy while reducing computational costs, a challenge that is also relevant to recognizing German Sign Language gestures [1,10,11,12].

The meta-analysis provided in [5] recognizes seven different classification approaches in use, namely *K*-nearest-neighbor-based approaches, support vector machine-based approaches, Hidden-Markov-Model-based approaches, artificial neural network-based approaches, CNN-based approaches, LSTM-based approaches, and other proposed approaches. The *K*-nearest neighbor-based approaches are supervised learning methods commonly used in sign language recognition. To classify the input data, the assumption is made that *K*-nearest data points are found, and then the input data are classified according to the dominant class among these neighbors. The *K*-nearest neighbor (kNN) classifier can be used in the field of EMG signals to categorize different types of movement according to the collected EMG data. Studies using kNN, such as those by [13] in French Sign Language (FSL) and by [14] on German Sign Language (GSL), have reported an accuracy ranging from 90% to 96.31%.

Support vector machine-based approaches have gained popularity recently in EMG pattern recognition. Even with a small number of training samples, SVM provides a reliable classification technique that performs well with high-dimensional data and achieves high classification accuracy. As a result, issues related to sign language recognition, including high-dimensional data and a small number of training examples, are well suited to SVM. Notable achievements include the accuracy of 90% achieved by [15] in recognizing words in the Indian Sign Language and the actual recognition of 80 words in American Sign Language (ASL) by [15] with an accuracy of 96.16%. Ref. [16] developed a system recognizing 50 ASL words using SVM, achieving an accuracy of 33.66%. This study highlighted validation techniques, including five-fold cross-validation and holdout validation.

Hidden-Markov-Model-based approaches combined with EMG signals have been effective in sign language recognition. A Hidden Markov Model (HMM) is a type of Markov model in which the observed data are influenced by an underlying unobservable Markov process. In an HMM, there is an observable process, and the outcomes of the said process are determined by the outcomes of the phenomena studied in a predetermined manner. Given that the studied phenomena are not directly observable, the objective is to gain insights into their state by observing the results of the observable process. Studies [17,18,19] reported an accuracy ranging from 86.7% to 96.5% for the Chinese and Brazilian sign languages.

Artificial-neural-network-based approaches show promise in recognizing gestures in sign language. ANNs have several benefits, including their ability to handle ambiguous and unclear data, a critical feature in the processing of EMG signals. In addition, ANNs are known for their outstanding generalization skills. Amatanon et al. [20] achieved an accuracy of 97.1% for the Thai sign language using an ANN-based system. Gupta et al. [21] studied the Indian sign language, and reported an accuracy of 87.5% when combining the EMG and accelerometer data.

CNN-based approaches use CNNs, which are a particular kind of neural network that performs well in image classification tasks. They have been widely used in many applications, including image recognition, object detection, and facial expression identification. CNNs have also been applied to the field of electromyography for the purpose of hand gesture detection and recognition in sign language [18]. MyoSign system achieved 93.7% word precision for ASL using CNN, LSTM, and CTC. In the work published by the authors, the researchers recognized three manual signs and three handshape sign language motions using a scale average wavelet transform and a rudimentary CNN classifier. This method produced an average accuracy of 94%.

Long-Short-Term Memory-based approaches use a recurrent neural network variant, which is recognized for its proficiency in capturing extended dependencies within sequential data. In the realm of electromyographic signals, researchers have used LSTMs to analyze and categorize different patterns of movement. Sernani et al. [22] achieved a precision of 97% to recognize 26 Italian sign language letters using bidirectional LSTM.

Other proposed approaches include, for example, the system introduced by [23], which combines motion sensors and EMG for sign recognition, to reduce the computational load. Khan et al. [24] achieved an accuracy 81% for the Pakistani sign language using empirical mode decomposition (EMD) and linear discriminant classifiers. The preliminary findings of our research demonstrate that deep learning, particularly convolutional neural networks (CNNs), outperforms traditional machine learning algorithms in the interpretation of EMG signals for gesture classification. Our literature review, further supported by, highlights the potential of Long Short-Term Memory (LSTM) networks in similar tasks. Given the promising results from CNNs and LSTMs, future research will explore more sophisticated deep learning architectures, with a specific focus on LSTM networks for real-time applications. Preliminary tests confirm the greater suitability of deep learning for this domain, underscoring the need for continued research to optimize these models.

The current study focuses on the application of EMG signals acquired from two distinct systems, the BIOPAC MP36 and the MyoWare 2.0 sensors, to classify gestures from PSL. The novelty of this research lies in the data acquisition with two different EMG recorders, which improves the reliability of the findings by ensuring consistency under the same experimental conditions. Such an approach addresses a significant gap in the literature, where studies often rely on a single device or dataset, thereby limiting the generalizability of results. By comparing signals derived from both a high-end and a more cost-effective device, we aim to assess the efficacy of each in producing a robust classifier for PSL gesture recognition, contributing novel insights into the feasibility of using lower-cost EMG systems for this purpose.

This study aims to compare the classification performance of machine learning techniques using datasets acquired from two devices. Specifically, it examines the effectiveness of these systems in recognizing both everyday gestures and critical SOS signals in PSL and international gesture systems used to signal life-threatening situations. The creation of a database from two different systems represents a unique contribution, offering a direct comparison of gesture interpretation across diverse sensor platforms.

The primary novelty of this study is the development of a high-quality dataset comprising signals from two distinct EMG devices, enhancing the robustness and validity of the classifier’s performance. Furthermore, the evaluation of a low-cost sensor (MyoWare 2.0) to produce a viable classifier for PSL gestures, including internationally recognized distress signals, offers a novel perspective within this field of study. The results underscore the potential for integrating affordable EMG technology into broader gesture recognition systems, providing a cost-effective solution for real-world applications.

The purpose of the study was to create different models capable of predicting certain hand gestures based on the EMG recording database (CNN, kNN, decision trees, logistic regression and linear SVM) and to compare them. Section 2 provides an overview of the related work, Section 3 introduces the dataset, experiment setup, and signal processing. In Section 4, we describe the algorithms we used in this study and in Section 5 we present the results. Our findings, limitations, and recommendations for future research are presented in Section 6. The study is concluded in Section 7.

## 2. Related Work

The interest in supporting deaf people was an inspiration for Norbert Wiener, a mathematician and engineer known as the father of cybernetics [25]. As a result of his efforts, on the second day of March 1950, Helen Keller appeared in an MIT advertisement demonstrating Helen’s hearing glove [26]. This section synthesizes the information provided on various advances and methodologies in sensory substitution technologies for sign language recognition.

Junior et al. emphasized the role of surface electromyography (sEMG) in facilitating communication for deaf individuals through Sign Language Recognition (SLR) systems. Their study focused on recognizing gestures of the Brazilian Sign Language (Libras) alphabet using sEMG signals captured via a Myo™ armband. They explored various signal processing parameters such as segmentation, feature extraction, and classification, achieving remarkable accuracies up to 99% with optimal settings [19]. Ong et al. proposed a novel discriminative approach based on Sequential Pattern Trees for SLR, leveraging deterministic features from hand trajectories. Their method demonstrated superior performance compared to nondiscriminative models such as Markov models in different datasets, highlighting scalability and efficiency in large-scale applications [27].

Rozado et al. addressed the complexities of SLR using a Bayesian-like paradigm called hierarchical temporal memory (HTM), inspired by neocortical principles. Their extended HTM framework significantly improved recognition accuracy for Australian Sign Language, showcasing its potential as a bio-inspired solution for time-sensitive pattern recognition tasks in SLR [28]. Sharma et al. tackled the challenge of dynamic American Sign Language (ASL) recognition using 3D convolutional neural networks (CNNs). Their approach outperformed existing models with improved precision, recall and F-measure metrics in the Boston ASL Lexicon Video Dataset, demonstrating feasibility for real-time applications [12].

Rezende et al. contributed significantly by developing a comprehensive public database for Brazilian Sign Language (Libras), enhancing research capabilities in SLR with deep learning models. Their dataset and findings provide a crucial resource for benchmarking SLR algorithms and advancing the field [29]. Ding et al. proposed an algorithmic approach to model and recognize the fundamental components of ASL signs from video sequences, emphasizing the holistic interpretation of sign language gestures for accurate recognition [30].

Deora et al. presented a framework for recognizing Indian Sign Language gestures using PCA and neural networks, addressing the challenges posed by complex hand movements and overlapping gestures [31]. Flasinski et al. and Kapuscinski et al. introduced a syntactic pattern recognition method for PSL hand postures, demonstrating the robustness and discriminative properties of gesture recognition systems and helping deaf individuals in an office setting [32,33].

Naser et al. [34] presented a multi-layer neural network with an autoencoder that recognizes five hand gestures (fist, open hand, wave in, wave out, and double tap) from sEMG signals recorded with a Myo armband with an accuracy of 99.68%, 100%, and 99.26% during training, validation, and testing, respectively. Their proposed multi-layer neural network outperformed the K-nearest neighbor classifier that served as a reference (accuracy of 97%).

## 3. Materials and Methods

**Figure 1:**
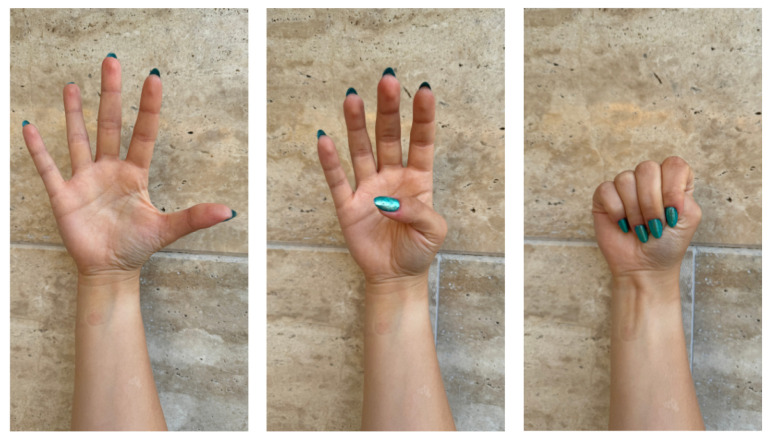
Gestures used during the experiment: steps of doing an SOS gesture.

**Figure 2:**
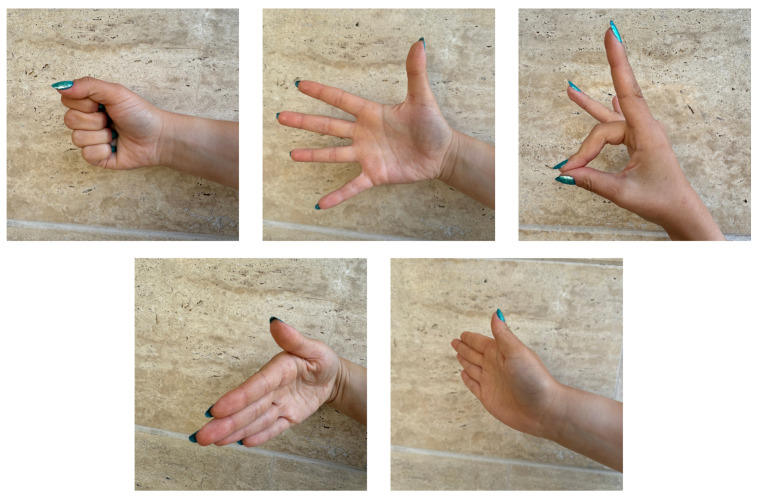
Gestures used during the experiment: game conrtol gestures.

**Figure 3:**
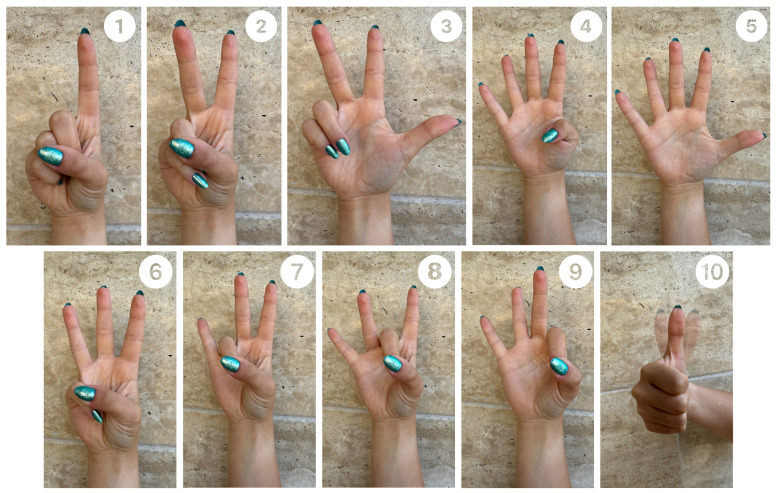
Gestures used during the experiment: numbers from 1 to 10.

**Figure 4:**
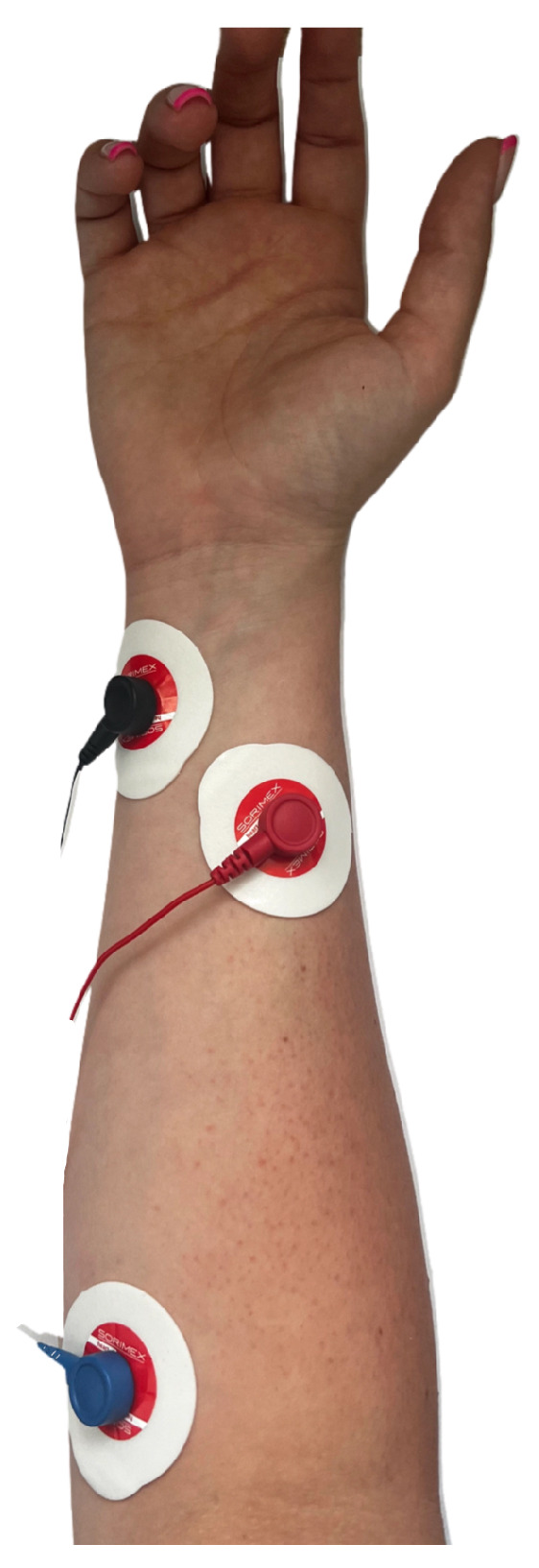
EMG electrode placement. Black, red, and blue cables are connected to the ground, positive input, and negative input, respectively.

Data were collected using the BIOPAC MP36 device (Biopac Systems Inc., Goleta, CA, USA) with Biopac Student Lab software and with the MyoWare 2.0 sensor (Advancer Technologies, LLC, Raleigh, NC, USA) at the Faculty of Biomedical Engineering. Eight students from the Faculty of Biomedical Engineering and Applied Mathematics participated in the EMG signal recordings. The study group consisted of 4 women (50% of the participants) and 4 men (50% of the participants), with an age range of 23–26 years (average age 23.88). The first round of studies was conducted using the BIOPAC MP36 system. Before starting the signal recording, volunteers were informed about the study procedures and practiced selected sign language gestures. The Polish Sign Language expressions considered included: grandfather [35], airplane [36], dragon [37], red [38], pitcher [39], water [40], apple [41], pear [42], emergency gesture (SOS signal; see Figure 1), fist, double tap, spreading fingers, wave left, and wave right (see Figure 2), and numbers from 1 to 10 (see Figure 3) [34,43,44]. The next step involved disinfecting the right forearm of the participant and connecting electrodes as shown in Figure 4.

**Figure 5:**
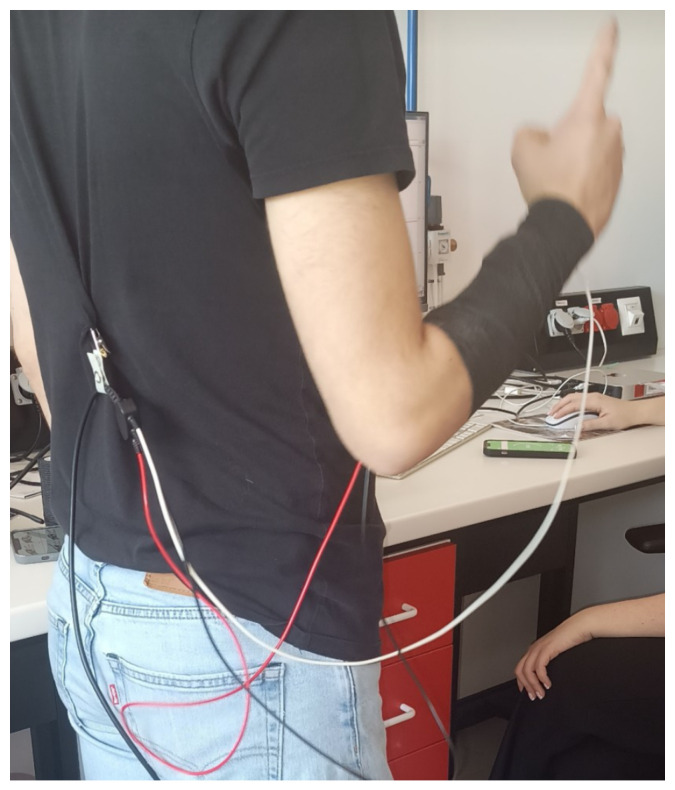
Taking measurements using the BIOPAC MP36 system and securing the electrodes with a cohesive bandage.

In addition, electrodes were secured with a cohesive bandage (see Figure 5) to minimize the risk of electrodes detaching during gesture performance. This approach also eliminated interference from moving cables. Measurements were made using the interval timer application. The recording began with a 10-s reference measurement, followed by a series of five repetitions of the selected gesture. Each participant performed two measurement series for each gesture. The time interval allocated to record one repetition was 5 s. A 1-s break between measurements was implemented. The total time to record one series was approximately 39 s. Measurements were carried out in a similar manner using the MyoWare 2.0 sensor.

### 3.1. Specification of the BIOPAC Measurement Device and Software

**Figure 6:**
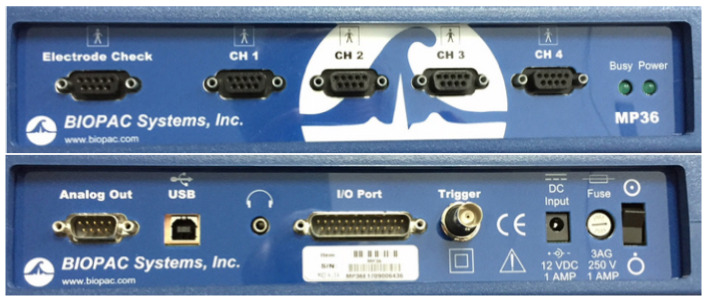
BIOPAC MP36 front and rear panels.

The BIOPAC MP36, along with the complete Biopac Student Lab system, is a solution that enables the conduct of lessons and scientific experiments. The MP36 is a data acquisition system that features an internal microprocessor that converts input data into a digital signal. The system converts the AC main power to DC, and the connection to the computer is made via USB 2.0. The MP36 allows for the reception of incoming signals, their transmission to the computer, the display of signals, and their storage in the computer’s memory. It has four input channels, but only one of them can function as a trigger input. Various devices, such as electrodes, transducers, headphones, or push-button switches, can be connected to the BIOPAC MP36. The front and rear panels of the MP36 are shown in Figure 6.

### 3.2. Specification of the MyoWare 2.0 Measurement Sensor

MyoWare 2.0 is a sensor that allows the measurement of muscle electrical potential, also known as EMG. As part of the project implementation, the entire MyoWare 2.0 ecosystem was purchased, consisting of snap-in connector system overlays. The modern snap-in connector system allows for easy and efficient connection of overlays, thus expanding the measurement system. The MyoWare 2.0 Muscle Sensor Development Kit includes [45,45]:

- 1× MyoWare 2.0 Muscle Sensor—muscle sensor—version 2.0.4;
- 1× MyoWare 2.0 Power Shield—power overlay;
- 1× MyoWare 2.0 LED Shield—overlay with segmented LED display;
- 1× MyoWare 2.0 Link Shield—MyoWare 2.0 muscle sensor adapter for Arduino;
- 1× 3.5 mm TRS audio cable—1 m long;
- 1× MyoWare 2.0 Arduino Shield—Arduino overlay;
- 1× USB A—USB C cable—2 m long;
- 1× Biomedical electrodes—10 pcs.;
- 1× MyoWare 2.0 Cable Shield—EMG sensor cable overlay;
- 1× Biomedical electrode cables;
- 1× MyoWare 2.0 Reference Cable—connection cable;
- SparkFun RedBoard Plus—Arduino-compatible development board.

**Figure 7:**
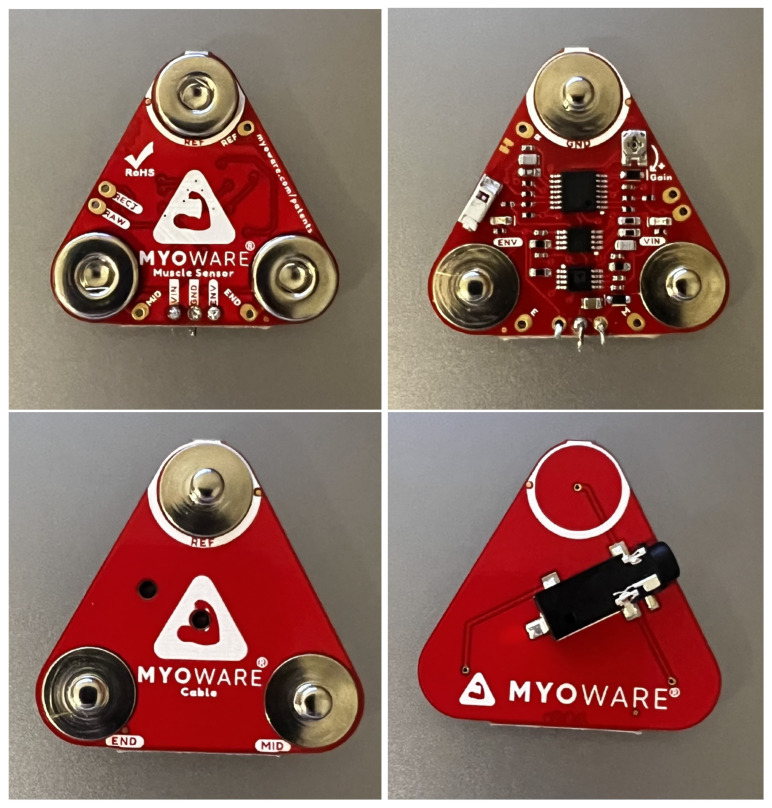

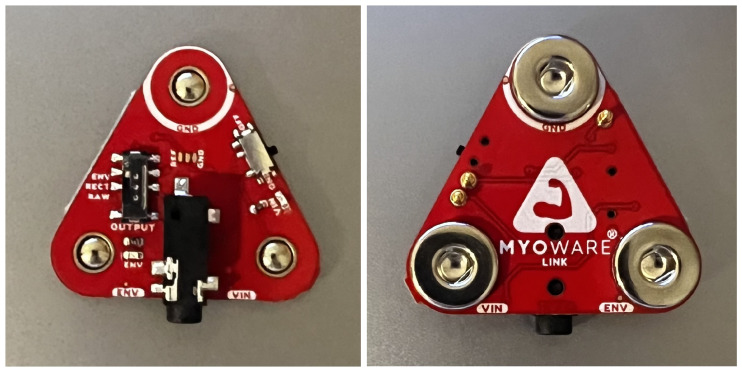
MyoWare 2.0 Muscle Sensor (first row), MyoWare 2.0 Link Shield (second row), and MyoWare 2.0 Cable Shield (third row).

**Figure 8:**
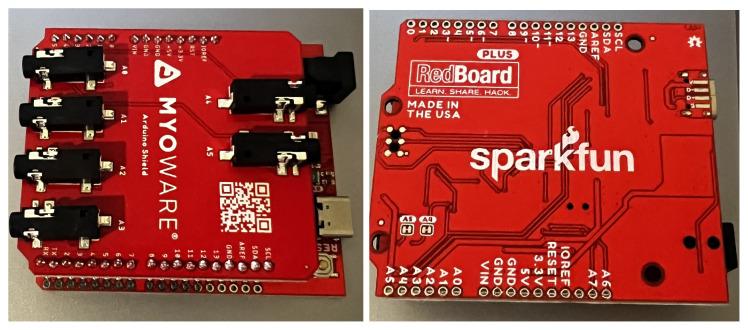
MyoWare 2.0 Arduino Shield + SparkFun RedBoard Plus.

**Figure 9:**
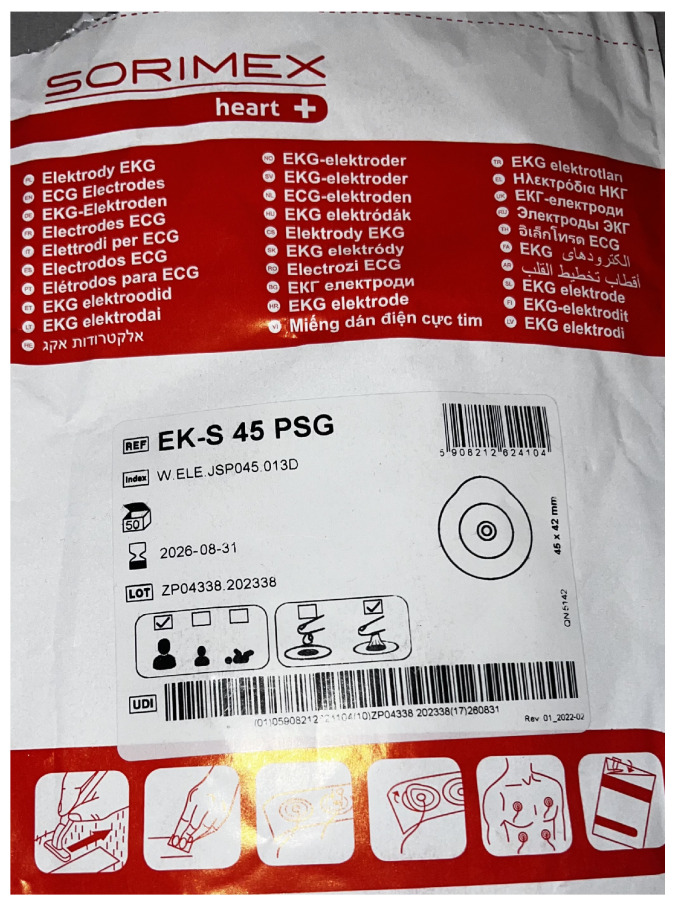
Biomedical electrodes SORIMEX heart+ EK-S 45 PSG.

To build the measurement module used in the project, the following components shown in Figure 7, Figure 8 and Figure 9 were used:

- MyoWare 2.0 Muscle Sensor—muscle sensor—version 2.0.4;
- MyoWare 2.0 Link Shield—MyoWare 2.0 muscle sensor adapter for Arduino;
- MyoWare 2.0 Cable Shield—EMG sensor cable overlay;
- MyoWare 2.0 Arduino Shield—Arduino overlay with SparkFun RedBoard Plus—Arduino-compatible development board;
- 3.5 mm TRS audio cable—1 m long;
- USB A—USB C cable—2 m long;
- Biomedical electrodes;
- Biomedical electrode cables.

**Figure 10:**
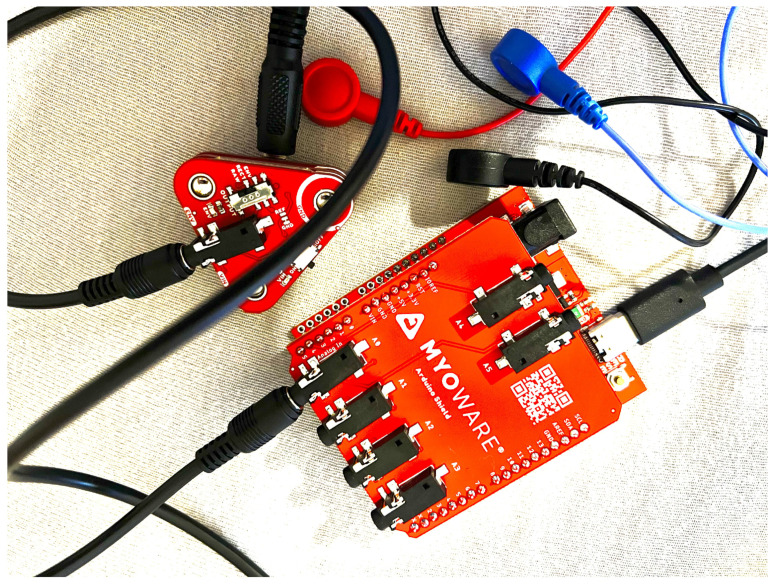
Measurement setup with a MyoWare 2.0 sensor.

The MyoWare 2.0 Muscle Sensor, MyoWare 2.0 Link Shield, and MyoWare 2.0 Cable Shield were connected with snap-in connectors. The MyoWare 2.0 Cable Shield was connected to the A0 analog input on the MyoWare 2.0 Arduino Shield using the TRS cable. Biomedical electrode cables were connected to the MyoWare 2.0 Link Shield and the biomedical electrodes, and the USB A–USB C cable was used to connect the system to a laptop. The setup is depicted in Figure 10.

Muscle potentials were recorded with software developed in the Arduino IDE (version 2.3.0) environment that connects to the MyoWare 2.0 sensor. The software registers and displays the values recorded in the analogue output A0, simultaneously indicating the beginning and end of the measurement cycle. The recorded signal is displayed on a single line, separated by a comma (,).

### 3.3. Signal Processing and Filtering

The signals were collected in folders whose names corresponded to the gesture. Preliminary signal processing was performed in the MATLAB environment. The program allows the user to select a folder and load the signals. Signal filtering was conducted to minimize disturbances and smooth out the drifting baseline. The first 10 s, which served as a reference, were removed from the signals. Subsequently, the signals were segmented into fragments related to respective gestures. Five-s segments were extracted from the signal with a 1.1-s break and saved in separate .csv files. In cases where a signal was slightly shorter for any reason, the record was padded with zeros. While simple and sometimes inaccurate, this method provided results that matched the expectations. Also, more complex methods were considered, such as resampling or using means of each record. Padding with zeros was sufficient since the tests were conducted on a database prepared by the research team. However, resampling will be risk-free and, overall, more accurate if one decides to interchange or merge databases. For each measurement after processing, a total of five .csv files were obtained, named according to the scheme: initials_gesture_name_measurement_number_gesture_number.csv, e.g., DD_1_1_gesture1.csv. The files prepared in such a way were stored in the database and were used as input for the classifier, bypassing the feature extraction process.

#### 3.3.1. Preprocessing Signals Obtained Using the Biopac System

**Figure 11:**
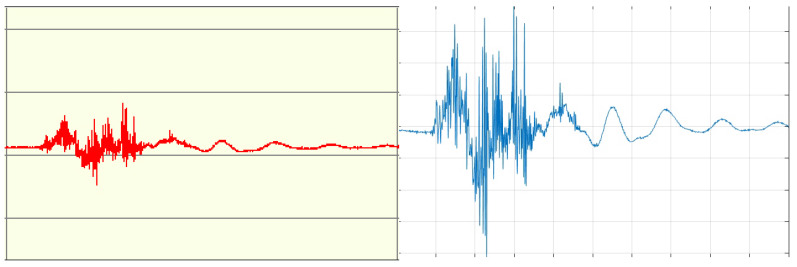
Example of a signal for the `double tap’ gesture obtained using the BIOPAC system (**left**) and generated in MATLAB (**right**). The x-axis is assigned to time and the y-axis is the signal amplitude.

Signals recorded using the BIOPAC system were loaded into the MATLAB environment and subjected to preprocessing. The signal sampling frequency was 1000 Hz. Figure 11 shows the signal for a gesture ‘double tap’ obtained using the BIOPAC system. The red graph represents the original signal, which can be modified using the virtual BSL analysis tools provided with the measuring device. The blue graph was generated in MATLAB.

**Figure 12:**
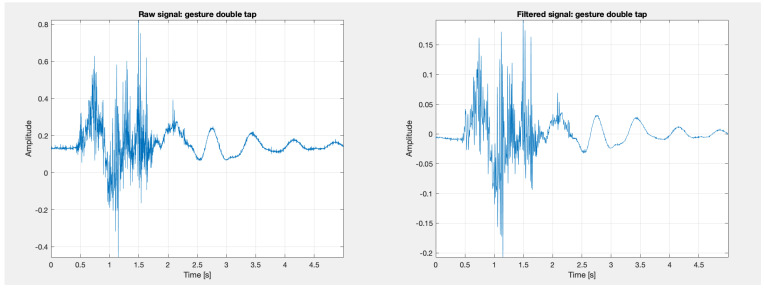
Example of a signal for the ’double tap’ gesture obtained using the Biopac system, processed in MATLAB.

Low-pass filtering with a cut-off frequency of 100 Hz and a filter order of 20, high-pass filtering with a cut-off frequency of 0.5 Hz and a filter order of 5, and band-pass filtering with lower and upper cut-off frequencies of 20 Hz and 450 Hz and a filter order of 20 were applied. A notch filter was also used to remove network interference, with cut-off frequencies of 49 Hz and 51 Hz. Examples of signals collected for the ‘double tap’ gesture are presented in the Figure 12. After filtering, the first 10 s of the signal (reference) were removed and the remaining signal was divided into five segments, each corresponding to a 5-s gesture.

#### 3.3.2. Preprocessing Signals Obtained Using the MyoWare System

**Figure 13:**
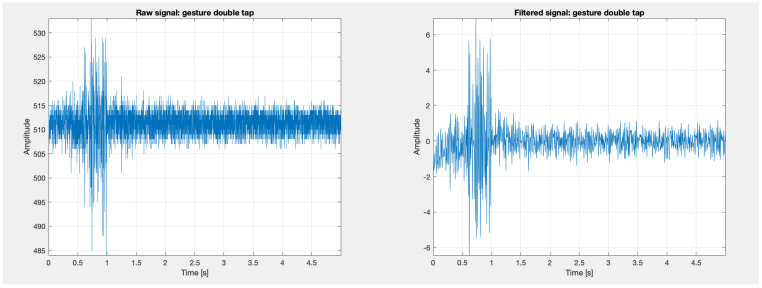
Example of a signal for the ’double tap’ gesture obtained using the MyoWare system, processed in MATLAB.

Signals recorded using the MyoWare system were loaded into the MATLAB (version R2023a) environment and preprocessed. The signal sampling frequency was 1000 Hz. Low-pass filtering with a cut-off frequency of 100 Hz and a filter order of 20, high-pass filtering with a cut-off frequency of 0.5 Hz and a filter order of 5, and band-pass filtering with lower and upper cut-off frequencies of 20 Hz and 450 Hz and a filter order of 20 were applied. A notch filter was also used to remove network interference, with cut-off frequencies of 49 Hz and 51 Hz. Examples of signals collected for the ‘double tap’ gesture are presented in Figure 13. The signal was then divided into five segments, each corresponding to a 5-s gesture.

## 4. Algorithms

Three algorithms were used during the analysis to provide comprehensive and varied results:

- K-nearest neighbors algorithm;
- Decision tree algorithm;
- Cross validation 5-fold neural network.

Although the kNN and the Decision tree classifier used were provided with the scikit-learn package, the 5-fold CNN (CNN, 5-fold) algorithm and the model used were created separately.

### 4.1. K-Nearest Neighbors

The kNN algorithm is one of the simplest supervised learning algorithms developed by Evelyn Fix and Joseph Hodges in 1951. It is based on evaluating the distance between the test sample and previously given training samples in a *n*-dimensional space (where *n* is the number of features in a dataset). After the distance is calculated, the algorithm considers *K* of the closest samples around the test sample and, based on the number of samples from different classes, predicts the sample class. The larger *K*, the more accurate the results will be [46].

Considering the kNN algorithm, one must take into account the metric used by the algorithm to determine the distance between samples. The most commonly used metrics are:

- Minkowski distance;
- Euclidean distance;
- Mahalanobis distance;
- Taxicab distance;
- Cosine distance.

#### 4.1.1. Minkowski Distance

The Minkowski distance is defined as:

(1) $$d_{st}=\sqrt[{p}]{\sum_{i=1}^{n}{\left|x_{si}-y_{ti}\right|}^{p}}.$$

Depending on the value of *p*, the distance can be interpreted as:

For p=1 — the taxicab distance;
For p=2 — the Euclidean distance;
For p⟶∞ — the Chebychev distance.

#### 4.1.2. Euclidean Distance

The Euclidean distance is defined as:

(2) $$d_{st}^{2}=\left(x_{s}-y_{t}\right){\left(x_{s}-y_{t}\right)}^{\prime }.$$

#### 4.1.3. Mahalanobis Distance

The Mahalanobis distance is defined as a distance between a data distribution and a point. It is defined as:

(3) $$d_{st}^{s}=\left(x_{s}-y_{t}\right)C^{-1}{\left(x_{s}-y_{t}\right)}^{\prime }$$

where C is the covariance matrix.

#### 4.1.4. Taxicab Distance

A specific case of the Minkowski distance (for p=1). It is defined as:

(4) $$d_{st}=\sum_{i=1}^{n}\left|x_{si}-y_{ti}\right|.$$

#### 4.1.5. Cosine Distance

The cosine distance is based around an included angle between two points:

(5) $$d_{st}=\left(1-\frac{x_{s}y_{t}^{\prime }}{\sqrt{\left(x_{s}x_{s}^{\prime }\right)\left(y_{t}y_{t}^{\prime }\right)}}\right).$$

In our case, the Minkowski distance metric with p=2 (so a Euclidean distance) with varying number of neighbors to consider was chosen to be used, as well as the Cosine distance metric.

### 4.2. Decision Tree

**Figure 14:**
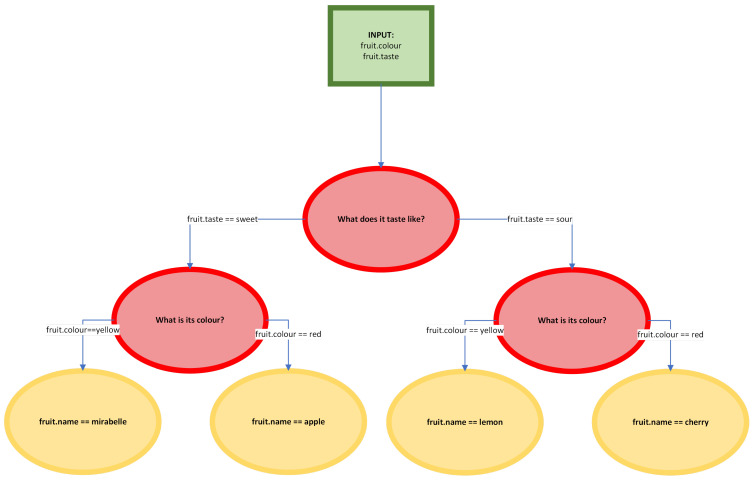
A simple representation of a decision tree.

The decision tree algorithm is a supervised learning classifier used in data mining and machine learning. The main premise of this classifier is the division between leaves, tests, and roots (see Figure 14). The classifier, using training data, can predict and assign a class to a test sample. Each class is a leaf—similarly to trees, the leaves mark an end of a branch. In this case, the branch is made up of multiple tests that classify the sample to a certain class.

### 4.3. Cross Validation Five-Fold Neural Network

The neural network model created consisted of three layers:

- Input dense layer;
- Hidden dense layer;
- Output dense layer.

#### 4.3.1. Activation

The first two layers used the Rectified Linear Unit activation, which uses a simple function to determine the output of a neuron:

(6) f(x)=max(0,x).

What this means is that if the value is negative, the function will output 0 and in any other scale it will output the given value. The greatest benefit of using ReLU activation is the nonlinearity it introduces, and the data outputted has a much more desirable gradient. The third dense layer uses Softmax activation, which computes the probability of a sample matching to each class (all probabilities sum up to 1). It is widely used in cases where the network is responsible for predicting the class to which the sample belongs.

#### 4.3.2. Neurons

Neurons are the most important element of the neural netework. The inspiration for them comes from the actual neurons found in every brain. All of them carry value and weight, and all operations performed within layers are based on those two features.

When a layer is dense, it means that all neurons are connected to each other. In the first layer, there are 32 neurons, in the second 64, and in the third, the number of neurons corresponds to the number of classes (in this case 14).

#### 4.3.3. How Do Neurons Operate?

1. The neuron computes its weighted sum:
  (7) $$z=\sum_{i=1}^{n}\left(w_{i}*x_{i}\right)+b,$$
  where *z* is the weighted sum, *w* is the weight of a neuron, *x* is its value, and *b* is bias (an additional value that might be given to a neuron to perform properly despite poor or improper input).
2. The output of the formula above is used as input in an activation function and its product is the final output of the neurons.

#### 4.3.4. *K*-Fold Method

The *K*-fold is a method of working with data that is based around a division of a dataset into *K* evenly sized groups. After that, each group is treated as a prediction group while the rest are treated as training data. This method provides the user with multiple outputs to work with, which allows for a better and more accurate evaluation of the model’s accuracy.

## 5. Results

Table 1 and Table 2 present the accuracy of different classifiers provided by the Classification Learner App, All-Quick-To-Train Algorithms, and a CNN and Table 3 shows CNN performance on data recorded with MyoWare and BIOPAC MP36, respectively. Table 4 summarizes the accuracy results of classifiers obtained in various research centres using the Myo armband (eight EMG sensors), where the classifiers were created using all five gestures presented in Figure 2 and Table 5 presents the accuracy of gesture recognition with MyoWare Muscle sensors using different algorithms.

The Classification Learner App has been part of MATLAB since version R2015a (MathWorks, Inc., Natick, MA, USA) [47]. The main purpose of using multiple different algorithms was to show the disproportion between neural network algorithms and supervised learning algorithms. Although the CNN algorithm takes the least amount of time to compute an output, it is much more accurate compared to the other classification methods used.

In Table 4, we show that deep neural networks (our proposed CNN and TL-augmented ConvNet proposed in [48]) are more accurate than other algorithms (original Myo system algorithm [49], K-nearest neighbor [49], and support vector machine [50]). That disproportion arises from the differences in complexity of classification algorithms and the fact that deep learning algorithms perfect their score over multiple epochs.

**Table 4:** Accuracy of algorithms on Myo armband data.

Method	Accuracy [%]	Reference
Myo system	83.0000	[49]
K-nearest neighbor	86.0000	[49]
Support vector machine	90.0000	[50]
TL-augmented ConvNet^1^	98.3100	[48]
Autoencoder	99.2600	[34]
CNN, 5-fold MyoWare	95.5307	This study
CNN, 5-fold BIOPAC	98.3240	This study

^1^ Convolutional neural network coupled with transfer learning.

**Table 5:** Accuracy of deep learning algorithms.

Method	Accuracy [%]	Reference
ANN	94.0000	[51]
Dense(32)		
LSTM(16)	98.5000	[52]
Dense(32)		
CNN, 5-fold	95.5307	This study

In addition, the CNN algorithm uses convolutional filters to capture patterns that improve signal recognition and classification. In training, these networks identify the most important features that improve accuracy. This process involves convolving filters with segments of input data to produce what is called a receptive field. Receptive fields allow individual filters to use the same weights for learning across all input segments. This field is then passed to the activation function [53].

## 6. Discussion

As part of the implemented project, a database of EMG signals corresponding to 13 gestures from Polish Sign Language and 11 gestures not originating from Polish Sign Language was created, with data obtained from the same research group using two different measuring devices the MyoWare 2.0 system and the BIOPAC system. A detailed analysis was carried out on the application of various learning algorithms, ranging from classic methods such as Decision Tree, KNN, and SVM, to advanced deep learning techniques, such as CNN.

The results of this study indicate that the convolutional neural network (CNN) algorithm outperformed the other classification methods in terms of accuracy when applied to the electromyographic (EMG) data recorded from both the BIOPAC and MyoWare 2.0 systems. Specifically, the CNN algorithm achieved an accuracy of 98.32% with the BIOPAC data and 95.53% with the MyoWare data, which is significantly higher than the accuracies obtained by the other algorithms, such as the Decision Tree and K-nearest neighbors (knn). This finding aligns with previous research that indicates that CNNs, particularly when combined with other deep learning techniques such as Long-Short-Term Memory (LSTM) networks or autoencoders, are highly effective in processing and classifying complex time series data, such as EMG signals [13,34].

The comparison between supervised learning algorithms (Decision Tree, KNN, SVM) and unsupervised learning algorithms (e.g., CNN) clearly demonstrated that unsupervised methods are superior in both accuracy and processing time for this type of data. The neural network models, although they require more computational resources and longer training times, provided much more reliable results. This is particularly important in real-time applications, where accuracy and speed are critical. These results echo the larger trend in machine learning, where deep learning techniques are increasingly favored for complex tasks involving high-dimensional data [28,34,44,48].

Comparisons of the proposed CNN algorithm with other custom built algorithms used in sign language recognition are difficult, since in this particular case, we proposed a custom-built database as a way to compare two recording devices. However, as seen in Table 5, the CNN algorithm related to this study achieved similar results to other algorithms used in different studies of the sign language recognition. During the evaluation of such algorithms, the secondary factor that should be taken into consideration is training and execution time. While the computation time of algorithms used in other studies is unknown, it is important to keep in mind that the dataset size might also differ from the one proposed in this paper.

Interestingly, despite the lower cost and complexity of the MyoWare 2.0 system, the accuracy achieved using this system was only slightly lower than that of the BIOPAC system. This suggests that with proper calibration and algorithmic support, lower-cost EMG sensors can be used effectively in applications such as sign language recognition. This is consistent with the findings of other studies that have successfully used lower-cost systems such as MyoWare in various gesture recognition tasks [19].

The novelty of our approach lies in the comparison of two different electromyographic (EMG) signal acquisition methods: the advanced BIOPAC MP36 system and the cost-effective MyoWare 2.0. Traditionally, most studies have relied on a single type of device, limiting the scope for comparison and generalization of results. By introducing two distinct systems, we enable a more comprehensive evaluation of gesture classification performance, while also assessing the potential for integrating lower-cost, more accessible EMG sensors with advanced deep learning algorithms. Our findings demonstrate that, despite its simpler design, the MyoWare 2.0 system achieves results comparable to the high-end BIOPAC MP36, paving the way for the adoption of affordable technologies in practical, everyday applications.

Although we demonstrate the effectiveness of CNN and highlight the potential of the MyoWare 2.0 system as a cost-effective alternative, there are several limitations compared to other studies in the field. The first limitation is a small and homogeneous study group consisting of eight subjects (four male and four female) between 23 and 26 years of age. The second limitation is the specific set of 24 gestures (described in detail in Section 3), including 13 from the Polish Sign Language. The third limitation is to use the BIOPAC and MyoWare systems with a specific configuration, which may not be optimal for all users or all types of gestures. Other limitations include the complexity of CNNs, the reliance on K-fold cross-validation, and the lack of real-world testing.

In contrast, other studies on sign language recognition, such as those using larger datasets from a wider range of users, have reported more robust findings that better account for inter-subject variability [29], explored a broader range of gestures or even full sign language lexicons, providing more comprehensive insights into the potential and limitations of EMG-based gesture recognition systems [12], determined the influence of different sensor placements and configurations on the accuracy and reliability of gesture recognition, sometimes finding significant improvements with alternative setups [19], examined more lightweight algorithms or hybrid models that balance accuracy with efficiency, particularly for mobile or embedded applications [20], implemented other validation techniques such as leave-one-out cross-validation or extensive testing on unseen data [18], and tested the system in real-world scenarios [28].

For future studies, we recommend recruiting a larger and more diverse study group, using entire corpora of sign languages and other gestures, alternative sensor configurations, and real-world testing.

## 7. Conclusions

This research has demonstrated the efficacy of convolutional neural networks in the processing and recognition of electromyographic signals for gesture identification, specifically within the context of Polish Sign Language. By using two distinct acquisition systems—BIOPAC MP36 and MyoWare 2.0—the study successfully highlighted the robustness of CNNs compared to traditional classification algorithms. While the BIOPAC MP36 system yielded slightly higher accuracy rates, the performance of the more cost-effective MyoWare 2.0 system remained commendably competitive, signifying its potential for broader applications. These results prove the viability of integrating affordable EMG-based technologies into gesture recognition frameworks, particularly for sign languages. Future research should aim to expand upon this work by exploring more diverse datasets, advanced deep learning architectures, and real-world applications, with the ultimate objective of refining the accuracy and accessibility of gesture recognition technologies. Moreover, the investigation into low-cost solutions paves the way for inclusive, scalable implementations in both healthcare and assistive communication domains. Furthermore, the findings reinforce the growing trend of using deep learning methods in signal processing tasks, particularly in contexts where accuracy and real-time processing are paramount. Future research should explore the potential for integrating more advanced deep learning architectures and investigate the feasibility of deploying such systems in mobile or wearable formats.

## Figures and Tables

**Table 1:** Accuracy scores of different methods on data recorded with BIOPAC MP36.

Model	Accuracy	Processing Time [s]
CNN, 5-fold	98.3240%	51.4798
Fine Tree	7.8571%	135.8637
Medium Tree	7.3214%	111.3142
Coarse Tree	6.4286%	69.4838
Fine kNN	7.0536%	78.7892
Medium kNN	6.6964%	139.4570
Coarse kNN	6.25%	152.1641
Cubic kNN	4.8214%	407.3334
Cosine kNN	7.0536%	202.6044
Weighted kNN	6.875%	221.3712
Efficient Logistic Regression	5.2679%	239.0899
Efficient Linear SVM	6.9643%	284.5885

**Table 2:** Accuracy scores of different methods on data recorded with MyoWare.

Model	Accuracy	Processing Time [s]
CNN, 5-fold	95.5307%	42.1773
Fine Tree	10.2679%	163.3825
Medium Tree	7.5893%	120.7889
Coarse Tree	6.5179%	82.5799
Fine kNN	10.1786%	94.6231
Medium kNN	5.7143%	223.3824
Coarse kNN	8.8393%	214.0173
Cubic kNN	6.25%	418.4289
Cosine kNN	5.9821%	208.4783
Weighted kNN	9.6429%	232.0177
Efficient Logistic Regression	9.6429%	244.6543
Efficient Linear SVM	9.6429%	276.6024

**Table 3:** Results of training the CNN network.

Statistic	MyoWare	BIOPAC MP36
Accuracy	0.0759	0.0580
Correct predictions	802	869
Incorrect predictions	94	27
Loss	12.4487	5.5726
Recall	0.0536	0.0313
F1 score	0.0725	0.0442
Precision	0.1121	0.0753

## Data Availability

The original data presented in this study are contained in the Supplementary Materials.

## Supplementary Materials

The following supporting information can be downloaded at: https://www.mdpi.com/article/10.3390/s24206710/s1, S1: EMG signal values for selected gestures.
